# Interploidy hybridization barrier of endosperm as a dosage interaction

**DOI:** 10.3389/fpls.2014.00281

**Published:** 2014-06-26

**Authors:** James A. Birchler

**Affiliations:** Division of Biological Science, University of Missouri-ColumbiaColumbia, MO, USA

**Keywords:** endosperm, dosage, imprinting, gene balance hypothesis, Dobzhansky-Muller incompatibility, small kernel effect, ploidy hybridization barrier, polyploidy

## Abstract

Crosses between plants at different ploidy levels will often result in failure of endosperm development. The basis of this phenomenon has been attributed to parental gene imprinting of genes involved with endosperm development but a review of the data from maize indicates a dosage interaction between the contributions of the female gametophyte and the primary endosperm nucleus to early endosperm development. However, it is noted that parental imprinting is a non-mutational means that can alter dosage sensitive factors and therefore can contribute to this effect. Operationally, the genes determining ploidy hybridization barrier would qualify for Dobzhansky-Muller incompatibilities that prevent gene flow between species.

The endosperm is a nutritive tissue in angiosperms that results from the fusion of one of the two sperm involved in double fertilization with the central cell of the megagametophyte (Birchler, 1993). In many species it is consumed before seed maturation but is persistent in the grains and hence its value for human nutrition. It has long been recognized that interploidy crosses would result in failure of endosperm development and hence seed abortion (**Figure 1A**). The basis of this phenomenon has been a matter of debate but operationally it serves as a hybridization barrier between any polyploid and its diploid progenitor(s), at least in many plants.

**FIGURE 1:**
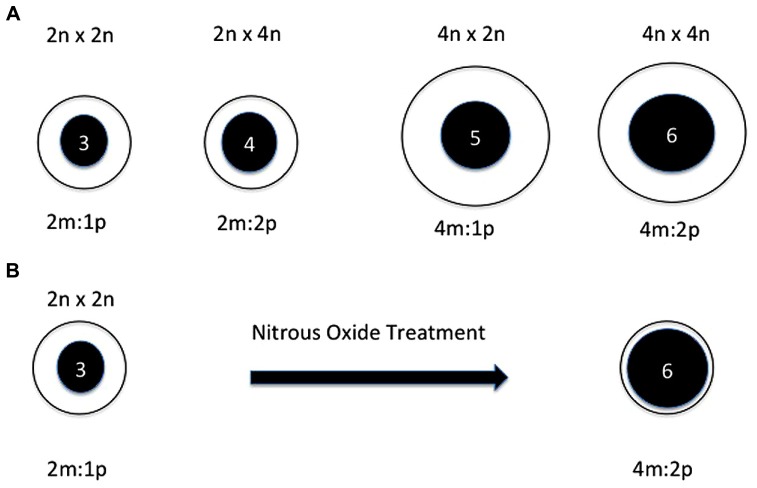
**(A)** Depictions of the primary endosperm cell after fertilization of normal and interploidy crosses. From left to right: diploid, interploidy diploid by tetraploid, interploidy tetraploid by diploid, tetraploid. The crosses are noted across the top. The cell size is depicted by the clear circles and the nucleus size is depicted by the inner filled circles. The ploidy of each nucleus is noted within and the maternal to paternal genomic relationship is shown below each image. The interploidy endosperms both result in developmental failure. In the diploid and tetraploid crosses the maternal to paternal relationship is the same as is the maternal gametophytic relationship to the genomic dosage after fertilization. In both of the interploidy crosses, the maternal to paternal contributions are changed as is the maternal gametophytic relationship to the genomic dosage after fertilization. **(B)** Depiction of the primary endosperm cell before and after chromosome doubling via nitrous oxide treatment. At the left is depicted a primary endosperm cell from a normal diploid plant. At the right is shown a depiction when the nucleus is doubled in chromosome number from triploid to hexaploid in the progenitor smaller cell. In the latter, even though the maternal to paternal relationship is the same as a tetraploid, the maternal gametophytic contributions would be typical of a diploid but with a doubled genome with which to interact. Based on a figure from Bauer (2006).

One idea to explain the evolution of interploidy hybridization barrier involves parental conflict with regard to resource allocation to the progeny (Haig and Westoby, 1989). The concept is that select genes involved with resource development will be imprinted from one parent or the other in such a manner that a maternal parent will optimize resources to all progeny but any one paternal parent, when there are different ones, will optimize the resources for his own progeny over other potential fathers.

The phenomenon of imprinting of individual genes was discovered by Kermicle in his analysis of the maize anthocyanin gene, *r1* (Kermicle, 1970). It exhibits full color when transmitted through the female but a mottled expression across cells when transmitted through the male parent regardless of dose. Imprinting has also been attributed to endosperm size factors that are found when chromosomal segments are missing from the sperm (Lin, 1982); however, we revisit this interpretation below.

Endosperm size factors refer to the situation that occurs with some translocations between the supernumerary B chromosome and normal A chromosomal segments in maize (Birchler and Hart, 1987). The B chromosome is basically inert and is neither required nor detrimental to plants possessing them unless their numbers exceed about 15 copies. It is maintained in populations by an accumulation mechanism that consists of nondisjunction at the second pollen mitosis, which makes the two sperm, and then preferential fertilization of the egg by the sperm that has the two B chromosomes. Thus, translocations between the B chromosome and the A chromosomes will have nondisjunction of the chromosomal segment attached to the B centromere. When some B-A translocations are used as a male parent, the progeny missing a paternal contribution to the endosperm are smaller than normal siblings (Roman, 1947; Lin, 1982; Birchler and Hart, 1987).

Several regions of the genome will produce this effect to a greater or lesser degree including 1S, 1L, 4S, 5S, 7L, and 10L being the most prominent in most backgrounds (Birchler and Hart, 1987). At least for the effect of 1L and 10L, there is evidence that the effect is cumulative from several regions that contribute to the whole arm impact (Lin, 1982; Birchler and Hart, 1987).

The argument that this small kernel effect is a reflection of imprinting was that for 10L, introduction of extra copies through the female parent was not observed to have any effect nor could it rescue the absence of the paternal copy (Lin, 1982). While this is the case for 10L in some backgrounds, extra copies of other chromosome arms introduced through the female parent does not rescue but enhances the paternal small kernel effect (Birchler and Hart, 1987). Indeed, the specific region of 10L that itself is responsible for a small kernel effect will enhance the analogous effect of 1S when transmitted in extra dosage through the female parent. This observation suggests that these genes function when passed through the female (including in the zygote) or at least in the female gametophyte.

Moreover, by crossing B-A translocations among themselves, it was realized that the same arms that produce the paternal effect would enhance that response of other arms when present in extra copy through the female parent (Birchler and Hart, 1987). Indeed, in the author’s materials, self pollination of the 10L translocations produce an additional class of further reduced sized kernels compared with crossing the translocation to normal females―just as occurs with all other regions of the genome that produce the small kernel effect. This result suggests that the responsible loci are in fact expressed when transmitted through the female parent and that they are involved in the same developmental process. The reason why this experience differs from Lin’s results is unknown but would seem to reflect background effects. Furthermore, the enhancement results indicate that these factors operate in a dosage sensitive manner and also that the stoichiometry of one region relative to others would magnify the effect. It was postulated that the developmental program established in the female gametophyte has a quantitative component that interacts with the dosage of the primary endosperm nucleus following fertilization (Birchler and Hart, 1987; **Figure 1A**).

Indeed, if one postulates that the small kernel effects are due to the absence of a gene that is normally expressed from only the paternal allele, then no such hypothetical gene could be vital because no known region of the maize genome is lethal to the endosperm when missing in the sperm. The maternal enhancement seems unlikely to result from imprinting because it does not involve an all or none contribution from the female parent but rather the number of copies transmitted. Moreover, there is no apparent impact on endosperm growth unless the same or other regions of the genome are paternally absent. Also, it is important to note that there are many mutations that when homozygous recessive are highly defective to the endosperm but no paternal absence in the genome has any such effect suggesting that none of these genes are expressed exclusively from the paternally derived allele. These considerations point to a quantitative explanation for the small kernel effects.

Many years later, studies of tetraploid formation suggested a related explanation of the interploidy disruption of endosperm development. Kato and Birchler (2006) produced tetraploid derivatives of several inbred lines of maize by treating self-pollinated diploid plants with nitrous oxide gas, which causes chromosome doubling, at about the time of the first mitotic divisions after fertilizations. Interestingly, the kernels on the ears of such treated plants have many defective endosperms. If the endosperms were doubled in ploidy, there would be no change in maternal/paternal genomic relationship but rather a change of the quantitative relationship of the maternal gametophytic gene products to the copy number of genomes in the now doubled primary endosperm nucleus (**Figure 1B**).

Bauer (2006) examined this phenomenon in more detail. Treatment of pollinated diploid plants near the timing of the first endosperm mitosis produced numerous defective endosperms. Cytological analysis indicated doubling of chromosome numbers to 60 chromosomes in the defective kernel class although some defective kernels were doubled twice or three times to yield ~120 or ~240 chromosomes, respectively. A time course of nitrous oxide treatment revealed very little effect at 12–17 hours after pollination (HAP), which immediately precedes the first division in the endosperm. The percentage of defective endosperms with elevated ploidy increased progressively with treatments at 14–19 HAP, 16–21 HAP and 20–15 HAP and then sharply decreased with treatment at 24–29 HAP. The latter result is not due to a failure of the action of nitrous oxide because at this timepoint, there is a sharp increase in the number of normal-sized hexaploid endosperms at the expense of normal-sized triploid endosperms. The results are consistent with the interpretation that genome doubling to produce defective endosperms has an early developmental window.

It should be noted that interploidy crosses have multiple variables that complicate their interpretation. They vary the maternal to paternal genomic ratios within the endosperm itself but they also vary the contribution of female gametophyte contributions to the number of genomic targets after fertilization. The chromosome doubling experiment separates these variables. The maternal to paternal ratio within the endosperm is maintained but the relationship of the maternal gametophytic contributions to the genomic targets is altered suggesting this possibility as the basis of the endosperm failure.

The timing of this relationship can be further deduced based on the behavior of B-A translocations after fertilization (Birchler, 1980). At a low frequency, B-A chromosomes are lost during development of the endosperm. If they carry an anthocyanin pigment marker, such loss can be readily recognized as a mosaic kernel. For the regions of the genome that produce the small kernel effect when there is no paternal contribution to the endosperm at all, there is no detectable effect in these mosaic kernels even for those with loss at early divisions (Birchler, 1980). These observations indicate that the maternal/primary endosperm relationship is critical at the initiation of endosperm development but not shortly thereafter. This nonautonomy could potentially be a reflection of the syncytial nature of early endosperm development but this consideration does not rule out the noted critical relationship. It should be noted that the syncytial nature of early endosperm development does not obscure the mosaic pattern of the anthocyanin marker or other mosaicism that results from transposable element action or chemical or irradiation mutagenesis of pollen.

Given that the interploidy endosperm failure can be mimicked by changing the maternal/zygotic dosage without changing the maternal/paternal relationship and the small kernel effect of paternal absence of regions of the genome is enhanced by maternal increase might suggest that segmental or genomic relative dosage might be responsible. These effects might not necessarily be the result of parental imprinting. However, some factors involved with early endosperm development, which have been identified in Arabidopsis, are in fact expressed only from alleles originating from one parent but not the other (Dilkes and Comai, 2004; Kradolfer et al., 2013). But imprinting results in a type of dosage effect and so an entanglement of interpretations is potentially possible. Dilkes and Comai (2004) have discussed development of the endosperm and how differential resource allocation among different paternal parents seems unlikely. It is potentially the case then that imprinting is a non-mutational process of manipulating the dosage of genes (Beaudet and Jiang, 2002) involved with endosperm development (Kradolfer et al., 2013). Those that exhibit imprinting that are involved with endosperm development are likely a subset of the whole group of genes that affect this process. Clearly, genes can be imprinted in the endosperm that have no impact on kernel size (e.g., the *r1* locus), which fact might also be taken to suggest that imprinting and resource allocation are not necessarily connected. Indeed, endosperm size is basically determined maternally; there is no perceptible difference in size when pollen parents from lines with very different endosperm sizes are used onto a common female line. Thus, there is no evidence in maize to suggest that the paternal parent has any influence on endosperm resource allocation.

What then is the driving evolutionary force for ploidy hybridization barrier in the endosperm? There may be none: it might simply be a neutral reflection of developmental and gene regulatory processes that have dosage components. However, operationally, it might serve to prevent the widespread occurrence of triploids in populations if allotetraploids and the diploid progenitors were to hybridize. While such hybridization in itself would not be productive, the widespread occurrence of any resulting triploids that would hybridize with both tetraploids and diploids to produce many aneuploid progeny would likely disrupt the population fitness to a much greater degree. Individuals with an inability to produce triploids would have a higher reproductive fitness. Mechanisms that prevent triploid production would potentially be selected and were apparently established early in angiosperm evolution but, as noted, this might be a neutral reflection of developmental mechanism.

There is considerable evidence from many experimental avenues that genomic balance when upset can have detrimental effects on the phenotype (Birchler and Veitia, 2010, 2012). As described above, the small kernel effect and the endosperm doubling results are consistent with this type of stoichiometric relationship. Thus, the endosperm interploidy hybridization barrier is likely an extension of the genomic balance phenomena. Indeed, all other tissues show impacts of aneuploidy so it would be unusual if the endosperm did not: the small kernel effects are the likely manifestation.

It is known from work primarily in potato that the genes for interploidy barrier are multigenic and can be overcome with manipulation of genome dosage (Johnston et al., 1980; Johnston and Hanneman, 1982). With divergence of the quantitative expression of these genes, a hybridization barrier occurs without a change in ploidy. These cases might qualify as classical Muller-Dobzhansky species incompatibility genes (Dobzhansky, 1937; Muller, 1942) that are compatible within species but not between species (Kradolfer et al., 2013). Consequently, while the responsible genes might diverge in a neutral fashion, they serve de facto as an isolating mechanism between polyploids and related diploids.

Based on the evidence described above, we argue that the ploidy hybridization barrier and the small kernel effect from segmental paternal absence represent a type of dosage interaction between the maternal contributions from the female gametophyte and the genome or segmental dosage in the primary endosperm nucleus. Because this interaction has a stoichiometric character to its behavior, it is likely related to the basis of standard aneuploidy syndromes (Birchler and Veitia, 2012).

## Conflict of Interest Statement

The author declares that the research was conducted in the absence of any commercial or financial relationships that could be construed as a potential conflict of interest.

## References

[B1] Bauer M. J. (2006). *The interploidy hybridization barrier in Zea mays* L. Ph.D. Dissertation, University of Missouri-Columbia, Columbia, MO

[B2] Beaudet A. L. Jiang Y.-H. (2002). A rheostat model for a rapid and reversible form of imprinting-dependent evolution. *Am. J. Hum. Genet.* 70 1389–1397 10.1086/340969 11992247 PMC379123

[B3] Birchler J. A. (1980). On the nonautonomy of the small kernel phenotype produced by B-A translocations in maize. *Genet. Res.* 36 111–116 10.1017/S0016672300019716

[B4] Birchler J. A. (1993). Dosage analysis of maize endosperm development. *Annu. Rev. Genet.* 27 181–204 10.1146/annurev.ge.27.120193.001145 8122901

[B5] Birchler J. A. Hart J. R. (1987). Interaction of endosperm size factors in maize. *Genetics* 117 309–317 17246405 10.1093/genetics/117.2.309 PMC1203206

[B6] Birchler J. A. Veitia R. A. (2010). The gene balance hypothesis: implications for gene regulation, quantitative traits and evolution. *New Phytol.* 186 54–62 10.1111/j.1469-8137.2009.03087.x 19925558 PMC2858765

[B7] Birchler J. A. Veitia R. A. (2012). Gene balance hypothesis: connecting issues of dosage sensitivity across biological disciplines. *Proc. Natl. Acad. Sci. U.S.A.* 109 14746–14753 10.1073/pnas.1207726109 22908297 PMC3443177

[B8] Dilkes B. P. Comai L. (2004). A differential dosage hypothesis for parental effects in seed development. *Plant Cell* 16 3174–3180 10.1105/tpc.104.161230 15579806 PMC535866

[B9] Dobzhansky T. (1937). Genetics and the Origin of Species. New York: Columbia University Press

[B10] Haig D. Westoby M. (1989). Parent specific gene expression and the triploid endosperm. *Am. Nat.* 134 147–155 10.1086/284971

[B11] Johnston S. A. den Nijs T. P. M. Peloquin S. J. Hanneman R. E. Jr (1980). The significance of genic balance to endosperm development in interspecific crosses. *Theor. Appl. Genet.* 57 5–9 10.1007/BF00276002 24302359

[B12] Johnston S. A. Hanneman R. E. Jr (1982). Manipulations of endosperm balance number overcome crossing barriers between diploid *Solanum*species. *Science* 217 446–448 10.1126/science.217.4558.446 17782980

[B13] Kato A. Birchler J. A. (2006). Induction of tetraploid derivatives of maize inbred lines by nitrous oxide gas treatment. *J. Hered.* 97 39–44 10.1093/jhered/esj007 16394254

[B14] Kermicle J. L. (1970). Dependence of the R-mottled phenotype in maize on mode of sexual transmission. *Genetics* 66 69–85 17248508 10.1093/genetics/66.1.69 PMC1212486

[B15] Kradolfer D. Wolff P. Jiang H. Siretskiy A. Kohler C. (2013). An imprinted gene underlies postzyogtic reproductive isolation in *Arabidopsis thaliana*. *Dev. Cell* 26 1–11 10.1016/j.devcel.2013.08.006 24012484

[B16] Lin B.-Y. (1982). Association of endosperm reduction with parental imprinting in maize. *Genetics* 100 475–486 17246065 10.1093/genetics/100.3.475 PMC1201823

[B17] Muller H. J. (1942). Isolating mechanisms, evolution and temperature. *Biol. Symp.* 6 71–125

[B18] Roman H. (1947). Mitotic nondisjunction in the case of interchanges involving the B-type chromosome in maize. *Genetics* 32 391–409 17247252 10.1093/genetics/32.4.391 PMC1209386

